# Digitized HIV/AIDS Treatment Adherence Interventions: A Review of Recent SMS/Texting Mobile Health Applications and Implications for Theory and Practice

**DOI:** 10.3389/fcomm.2020.530164

**Published:** 2020-11-10

**Authors:** Lunthita M. Duthely, Alex P. Sanchez-Covarrubias

**Affiliations:** 1 Obstetrics, Gynecology and Reproductive Sciences, Division of Research and Special Projects, University of Miami Miller School of Medicine, Miami, FL, United States; 2 Obstetrics, Gynecology and Reproductive Sciences, Division of Gynecologic Oncology, University of Miami Miller School of Medicine, Miami, FL, United States

**Keywords:** access to healthcare, mHealth ethics, minorities, health disparities in HIV/AIDS, intervention - behavioral, HIV/AIDS, mHealth

## Abstract

**Background:**

Mobile health technologies (mHealth) are efficacious along the continuum of HIV/AIDS—from prevention of HIV transmission to those at the highest risk of acquiring infection, to adherence to HIV medical care, for those living with the disease—decreasing the public health burden of the disease. HIV/AIDS is a complex condition, as certain population subgroups are disproportionately affected. Furthermore, barriers experienced at the individual level (e.g., HIV stigma) and at the systems level (i.e., access to care) contribute to these disparities. Low cost, high penetration rates and ease of use mean mHealth SMS/texting solutions hold the biggest promise for curbing the global HIV/AIDS epidemic; yet these technologies have their own challenges. Our primary objective was to assess interventions that promote adherence, which are delivered via SMS/texting, and important design and ethical considerations of these technologies. Specifically, we evaluated the underlying frameworks underpinning intervention design, strategies to safeguard privacy and confidentiality, and measures taken to ensure equity and equitable access across different subgroups of persons living with HIV (PLWH). We also synthesized study outcomes, barriers/facilitators to adherence, and barriers/facilitators of technology to support HIV adherence.

**Methods:**

A scoping review methodology was utilized, searching the Medline database for recently published articles (January 2017 to June 2019). Two reviewers independently screened titles and abstracts for relevancy using the following eligibility criteria: (a) original research or protocol; (b) inclusion of persons living with HIV; (c) intervention delivery via SMS/text messaging; and, (d) intervention included HIV care adherence.

**Results:**

Seven (7) of the 134 articles met full criteria. The great majority (n = 6) did not report whether the interventions were developed under established behavioral change models or frameworks. Strategies to address privacy, confidentiality and equity/equitable access were taken in four (*n* = 4) studies.

**Conclusion:**

Our mixed methods review determined that privacy and confidentiality remain a concern for PLWH. Provisions to accommodate literacy, infrastructure, technology and other challenges (e.g., access to smartphones and Wifi) are important ethical considerations that guarantee equity and equitable access. Further investigation will determine the contexts within which theoretical models and frameworks remain relevant in the rapidly evolving field of digitized interventions that support adherence.

## BACKGROUND

### Introduction

Recent reviews have demonstrated the potential for mHealth SMS/texting to promote adherence among people living with HIV (PLWH) (Daher et al., 2017; Mayer and Fontelo, 2017; Muessig et al., 2017; Purnomo et al., 2018; Shah et al., 2019). Digitized interventions, which include medication alerts, appointment reminders and behavioral interventions that address individual-level barriers care (Henny et al., 2018) are explored increasingly in large clinics, rural or remote regions and other resource-poor environments (Jack and Mars, 2014). There is, however, a limited body of knowledge on ethical considerations in the design and deployment of these interventions (Perez et al., 2015). We therefore embarked on a scoping review of recent HIV mHealth applications delivered via SMS/texting. Taking into consideration that the development of an intervention requires a solid literature review with reference to relevant theories (Evans et al., 2016), we aimed to answer if there are established frameworks or models that underpin the design of mHealth interventions? There is also increasing evidence that privacy and confidentiality considerations may be inadequately addressed for these interventions (Jack and Mars, 2014), which led us to determine what design strategies are implemented to address privacy and confidentiality for those who use mHealth applications?

Finally, an effective intervention will meet the needs of their intended population equally (Evans et al., 2016; Amankwaa et al., 2018). The specific context of the intended users, women in the developing world, for example (Duggal et al., 2018), who may be marginalized due to their status in society, is an important consideration when designing interventions as well. Therefore, we also aimed to evaluate whether measures were taken to ensure equity and equitable access across all users of the mHealth application.

### HIV/AIDS: A Chronic Condition—A Complex Condition

HIV/AIDS is a complex condition. On the one hand, the advent of combination anti-retroviral therapy (cART) allows an HIV/AIDS diagnosis to be managed increasingly as a chronic condition; on the other hand, barriers to adequate HIV care result in disparate outcomes for those living with HIV/AIDS (White House Office of National AIDS Policy, 2016; Crepaz et al., 2020; Essuon et al., 2020). It is well-documented that HIV/AIDS is a highly stigmatized condition (White House Office of National AIDS Policy, 2016; Drysdale et al., 2020), and PLWH, who are more likely to be diagnosed with other stigmatized conditions—e.g., poor mental health status, psychiatric disorders, substance use disorders—may be further stigmatized because of these negatively viewed, compounding conditions, which interfere with adherence to care (White House Office of National AIDS Policy, 2016; Centers for Disease Control, 2018; Sullivan et al., 2019).

### Curbing and Ending the HIV/AIDS Epidemic

HIV/AIDS is a global public health problem, which is being addressed internationally on multiple fronts. The joint United Nations Program on HIV and AIDS (UNAIDS) targets to end the HIV/AIDS epidemic by 2030. The UNAIDS “90-90-90” goals, which were projected for the year 2020, state that 90% of PLWH will know their status (be tested), 90% of those who know their HIV-positive status will be on treatment, and 90% of those on treatment will have suppressed HIV viral load (The Joint United Nations Programme on HIV and AIDS [UNAIDS], 2015). The United Nations (UN) Sustainable Development Goals (SDGs) seek to end the HIV/AIDS epidemic by the year 2030 (United Nations, 2015). These goals are attainable when PLWH are on in HIV care and on their cART regimens.

Persons living with HIV who are engaged in care have improved health outcomes, including better viral suppression—which decreases the spread of HIV within the community (Okano et al., 2016). In fact, statistical models predict that engaging PLWH in primary health care will have the largest impact on reducing the global, public health burden of HIV/AIDS (Shah et al., 2016). Conversely, PLWH who are disengaged from HIV care struggle to maintain HIV viral suppression (viral suppression), and are more likely to transmit HIV to others (National Institute of Allergy and Infectious Diseases, 2018).

### HIV Outcomes Disparities

Worldwide, different subgroup populations—e.g., ethnic minorities, men who have sex with men (MSM), young women—are disproportionately affected by HIV/AIDS. Recently published examples are presented here. For many middle- to low-income countries and other resource-limited environments, HIV/AIDS services are limited and exacerbate negative health outcomes. In Asia, for example a lack of adequate mental health services (Li et al., 2013) affect urban-dwelling Chinese PLWH disproportionately (Guo et al., 2020). An inability to link to ART for injection drug users, older persons, and those engaged in sex work also affect PLWH in other parts of Asia (Alaei et al., 2018). HIV outcome disparities are prevalent in high income countries, as well. In Europe, the HIV epidemic is concentrated among MSM, who have lower rates of adherence to ART; this in turn has significantly increased the spread of the disease (Okano et al., 2016). In the U.S., epidemiological studies report that minorities and young women carry a higher burden of adverse health outcomes, which is unequal across age, gender and mode of acquisition (e.g., perinatally acquired HIV); those who acquired HIV perinatally had lower rates of viral suppression (Crepaz et al., 2020). African Americans in rural areas were less likely to be linked to HIV medical care, retained in care or virally suppressed, when compared to their white, non-Hispanic counterparts (Essuon et al., 2020). Cross-sectional studies have also reported these differences. Young adult (13 to 29 years old) African American women, for example, were found to have the lowest rates of sustained viral suppression (Dale et al., 2019).

### HIV Medication Adherence Challenges

Non-adherence to medications is of particular concern for those living with HIV, due to the deleterious consequences of not taking medications regularly to suppress the HIV virus. It has been demonstrated across different regions worldwide that systemic and cultural barriers affect medication adherence for different population subgroups. Among women, disengagement from HIV care leads to non-adherence to cART (Okawa et al., 2015) and detrimental health effects, including death (Watts et al., 2013). U.S. ethnic minority women report discrimination and gendered racial microaggressions (subtle forms of discrimination), which is directly related to adherence to care (Dale et al., 2019). Barriers to medication adherence include perceived stigma and fear of unintended disclosure of the HIV positivity (Madiba and Josiah, 2019). On the African continent, newly diagnosed pregnant women are more likely to be non-adherent to ARV than those with a known HIV status before pregnancy (Okawa et al., 2015)—demonstrating a need for interventions that target barriers to HIV care adherence.

### mHealth Interventions for PLWH

mHealth interventions have been efficacious along the spectrum of HIV/AIDS—including prevention of HIV transmission, early diagnosis and referral to treatment, and treatment of associated conditions for those in HIV care (Catalani et al., 2013; Ingersoll et al., 2015; Rana et al., 2016). Medication and appointment adherence is the target of most mHealth interventions (Bauermeister et al., 2017; Hightow-Weidman et al., 2018). mHealth is explored increasingly for PLWH for several reasons: limited health care service availability in resource-restricted or large clinical settings (Amankwaa et al., 2018), the ubiquitous nature of cell phone usage (95% mobile phone coverage in the world) (Sanou, 2016), and the importance to address both the mental health and medical needs of the individual living with HIV (White House Office of National AIDS Policy, 2016).

mHealth solutions are varied in complexity, and the research on HIV mHealth interventions is growing rapidly. mHealth interventions include-, but are not limited to-, interventions delivered telephonically, interventions delivered via SMS/texting, and full-blown applications (apps), which may be powered by artificial intelligence (Cole-Lewis and Kershaw, 2010). Apps include interactive communications, as well as gaming approaches. SMS/texting delivery systems are desired globally, due to the relatively low implementation cost and high penetration rates (Chan and Kaufman, 2011; Miller and Himelhoch, 2013; Amankwaa et al., 2018). Cell phone SMS/texting is the most widely used and inexpensive form of mobile communication, available to the most basic phones currently in use.

### Ethical Dimensions of mHealth Interventions

There are divergent views regarding both the benefits and drawbacks of digital approaches to health such as mHealth interventions (Marent et al., 2018a). Critiques can be summarized as: a lack of clearly defined models upon which interventions are based (Cole-Lewis and Kershaw, 2010; Labrique et al., 2013; Perez et al., 2015; Evans et al., 2016); “weak theoretical underpinning” of the interventions (Evans et al., 2016); and, (the lack of) ethical considerations for digitized interventions for PLWH (Labrique et al., 2013; Jack and Mars, 2014). Despite the documented successes, there is growing concern regarding the minimization or lack of clearly defined ethical principles when designing and deploying mHealth interventions (Labrique et al., 2013; Jack and Mars, 2014; Perez et al., 2015). The body of knowledge on the ethical considerations of these interventions lags behind the growth in mHealth applications, particularly as they apply to marginalized populations (Jack and Mars, 2014). As mentioned previously, HIV care is not equal for PLWH (White House Office of National AIDS Policy, 2016; Crepaz et al., 2020; Essuon et al., 2020). Perez et al. (2015) encourage designers of SMS/texting interventions to consider ethical principals in the design of mHealth interventions, and to abide by a set of principles to ensure safety and confidentiality of the intervention population. In summary, mHealth interventions designs should be built on a foundation of one or more theoretical models or frameworks, and privacy, confidentiality, equity and equitable access should be at the forefront of the mhealth design. We follow with an overview of theoretical models/frameworks, privacy, confidentiality, equity and equitable access as ethical dimensions of mHealth interventions.

#### Intervention Theories, Models, and Frameworks

Theories, models and frameworks are important considerations for digitized interventions. Health behavior models serve multiple purposes, from the design phase to the deployment phase of mHealth interventions (Burrus et al., 2018). They guide the development of mHealth interventions, which by nature can rapidly respond and adapt to end user and other inputs (Riley et al., 2011), and enhance impact and usage of digitized HIV interventions (Burrus et al., 2018). Involving users at the design phase may avert the drop off in usage that occurs over time (Schnall et al., 2015). Also, the model should be an appropriate model for the intended population. As in important ethical consideration, adolescents, for example, may have the technical ability to navigate a specific platform, yet certain models or frameworks may not be developmentally appropriate for that age group (Hightow-Weidman et al., 2015).

Here we list the more commonly cited face-to-face behavior theories that have been digitized, which we found in the literature: Self-Determination Theory (Deci and Ryan, 1995); Stages of Change (Rhodes and Malotte, 1996); Theory of Planned Behavior (Godin and Kok, 1996); Theory of Reasoned Action (Jessor and Jessor, 1977); Social Cognitive Theory (Bandura, 2001); and, the Health Belief Model (Glanz et al., 2004). It is not uncommon to find that more than one model of expected behavior change was applied to the mHealth design (Riley et al., 2011). One such example is gaming theory to effect health behavior change.

Gaming is often cited as a design feature for interventions designed for adolescents and young adults and the Information, Motivation, and Behavioral Skills (IMB) model (Amico et al., 2005) is one model that has been applied to gaming theory. The antecedents to health behavior change in IMB are being well- and accurately-informed, being motivated personally and socially to engage in the behavior, and having the appropriate skills and level of self-efficacy to apply the change(s) (Fisher et al., 2009). The IMB model, consistent with the social learning theory, is broadly applicable model, which has been used to develop and create theoretically-based gaming content (Whiteley et al., 2018).

#### Privacy and Confidentiality

Next, we review the ethical dimensions of privacy and confidentiality, as they relate to mHealth interventions for PLWH. For the purposes of this review, we defer to following U.S., South African and WHO definitions of privacy and confidentiality. Privacy and confidentiality are defined by the U.S. Department of Health and Human Services (2009) in the U.S. and by the National Department of Health (DoH) Ethics in Health Research Guidelines in South Africa (Staunton et al., 2019). Privacy is the right of an individual (e.g., the PLWH) to keep his / her information private from others, and not unlawfully collected or disseminated (U.S. Department of Health and Human Services, 2009; Staunton et al., 2019) and confidentiality is the protection of health information, which is entrusted to another entity (e.g., the owner of the mHealth SMS/texting system), is kept private (U.S. Department of Health and Human Services, 2009; Staunton et al., 2019). Another aspect of privacy is data privacy—the ability to guarantee that information and data about a person will be protected against both intentional and unintentional exposure (World Health Organization, 2015).

Historically, an HIV/AIDS diagnosis is a highly stigmatized condition in the U.S. (Earnshaw et al., 2013) and elsewhere (Purnomo et al., 2018), and PLWH experience discrimination because of this (Dale et al., 2019). HIV stigma, in turn, results in non-disclosure to family members, older children, and intimate partners (Labrique et al., 2013; Taylor et al., 2020) point to the introduced risks to those living with HIV/AIDS that could result in social marginalization, psychological stress, invasion of privacy or breach of confidentiality, due to a loss in privacy or confidentiality. Generally, these risks could be categorized as physical, social, behavioral, and psychological risks. Labrique et al. (2013) discuss the “fate of text (SMS) messages” (p. 5)—a text message could be unintentionally read by a different recipient. As an example, in resource-poor regions or rural areas, individuals may share phones or rely on others to communicate for them (Jack and Mars, 2014; Evans et al., 2016; Purnomo et al., 2018).

#### Equity and Equitable Access

The next issue to be considered is, whether the mHealth application considers equity and equitable access across different populations the applications are designed for. What ensures equity, maximum and equitable access? The World Health Organization’s (WHO) “Equity, Social Determinants and Public Health Programmes” report identified five major indicators of equity (World Health Organization, 2010): socioeconomic status, differential exposure, differential vulnerability, differential healthcare outcomes, and differential consequences.

Socioeconomic status, defined as a social position unique to individual cultural settings, is attributed to factors like gender, education, income and occupation (Dahlgren, 2006). Differential exposure, oftentimes related to social position, includes exposure to risk factors, such as barriers to adopting healthy behaviors (Evans et al., 2001). Differential vulnerability refers to increased difficulty, given the already disadvantaged state of an individual, as a result of a clustering of risk factors, such as low income and social exclusion (Evans et al., 2001). Differential healthcare outcomes, defined as disparities in healthcare access that are a result of health systems providing services that are less effective for certain population groups (Solar, 2007). Finally, differential consequences, which arise on a personal level as a result of the previously defined concepts. As explained by Solar (2007), differential health consequences are at the end of a chain of reactions triggered by differential social positions in society.

There are specific considerations with respect to equitable access for technology-infused, health-related communications. Equitable access would consider consistent cellular and Internet service; accommodations for those who many not have the economic means to meet the technical requirements; considerations for low-literacy individuals, who may rely on others to communicate for them; and, accommodations for low levels of health literacy or technology-related literacy (e.g., non-native speakers, the elderly) (Jack and Mars, 2014).

#### Aims and Objectives

In summary, digitized HIV interventions hold promise toward ending the HIV epidemic and require careful and ethical considerations. The complex mental health and medical needs of PLWH make mobile interventions, specifically, an attractive solution for limited resource settings (Amankwaa et al., 2018), because of the lower cost of these interventions, coupled with the high penetration rates of cell phone usage worldwide (Sanou, 2016). Mobile technologies are growing in use and effectiveness—matched with documented successes (Muessig et al., 2015; Taylor et al., 2019)—yet, require careful consideration. Interventions may not be theoretically based (Cole-Lewis and Kershaw, 2010; Labrique et al., 2013; Perez et al., 2015; Evans et al., 2016), may benefit certain subgroups disproportionately (Amankwaa et al., 2018), and the designs may face privacy and confidentiality challenges. Furthermore, dissemination and implementation barriers may prevent the successful utilization of these technologies for the intended population (Kempf et al., 2015). We, therefore, embarked on a scoping review, with the primary aim of addressing the following questions.

Research Questions:

1. Are mHealth interventions based on established frameworks or theories?
2. Are privacy and confidentiality addressed in mHealth designs?
3. Are populations for whom the interventions are designed considered equally?

The secondary aim was to report on study outcomes for the interventions we reviewed, and synthesize barriers and facilitators to HIV care adherence, and barriers/facilitators of technology to facilitate adherence to HIV care identified in the qualitative studies we reviewed.

## METHODS

We employed a scoping review methodology (Arksey and O’Malley, 2005). Scoping studies aim to rapidly map the key concepts underpinning a research area or questions and consider multiple sources and types of evidence available. Specifically, our review was guided by Levac et al. (2010) framework, which extended and elaborated on the original scoping review framework (Arksey and O’Malley, 2005).

The required 5 steps are outlined here:

1. State the research question(s) and rationale for conducting the study (see 1.9).
2. Identify studies: Work with a team that includes methodological expertise (see Information Sources).
3. Select studies: use an Iterative process with two researchers (see Information Sources and Search Process)
4. Charting the data: use an iterative process, where a qualitative content analysis is suggested (see Data Charting Process)
5. Numerical and thematic analysis: specify unit of analysis, include meaning of the findings related to study objective and implications for future research, practice or policy (see Results).

### Eligibility Criteria

A limited, mixed-methods review was conducted, where we searched and synthesized the most recent 2 years of publications available (2017 to 2019), to assess digitized interventions for HIV care adherence that are delivered via mobile SMS/texting.

The inclusion and exclusion criteria we applied to the articles are the following:

1. One or more of the components of the were delivered via a mobile device.
2. The intervention population included persons living with HIV.
3. At least one component of the mobile intervention was delivered via SMS/texting.
4. The study type was a protocol or a randomized controlled trial.
5. Interventions employing telephone calls, only, were excluded.

Since we aimed to understand HIV care interventions, only articles that described randomized controlled trials and protocol-type articles were included. Additional, reasonable steps were taken to identify related studies or online documentation regarding the technical aspects of the mHealth interventions we reviewed. As described previously, SMS/texting is the most widely used and inexpensive form of communication available to even basic mobile phones; we, therefore, limited our review to include only interventions with one or more components delivered via SMS/texting.

### Information Sources

Medline was the only database included in the study. In consultation with a biomedical librarian, a search strategy was developed to conduct a comprehensive search and identify recent and relevant studies of the database between 1/1/2017 and 2/28/2019. In line with several other recently published HIV mobile health reviews (e.g., Hightow-Weidman et al., 2015; Bauermeister et al., 2017; Muessig et al., 2017); we chose a 2 year time frame. As the field of mobile interventions for HIV care is rapidly evolving, we therefore extended the timeframe in a subsequent search to 6/30/2019. This allowed for the lag time (up to 12 months), from publication, to the time when MEDLINE information specialists apply MESH terms to published articles.

### Search Process

Appropriate MESH terms related to “HIV,” “AIDS,” “mHealth,” “mobile health,” and “interventions,” were identified by the biomedical librarian. The resulting PMID numbers were forwarded to the review team. Review team members, A.P.S. and L.M.D., Reviewer 1 and 2, respectively, screened and coded articles using a pre-defined set of procedures (see Selection of Sources of Evidence, Data Charting Process, and Data Items). The complete search strategy is documented in Supplementary File 1.

### Selection of Sources of Evidence

Reviewers 1 and 2 independently screened the article titles and abstracts for relevancy to the general topics of “HIV,” “AIDS,” and “mobile health.” Through an iterative process of three rounds of review, articles were coded initially by identifying the article type and technology mode. Reviewers consulted one another and came to a consensus to resolve coding discrepancies.

### Data Charting Process

**FIGURE 1 |:**
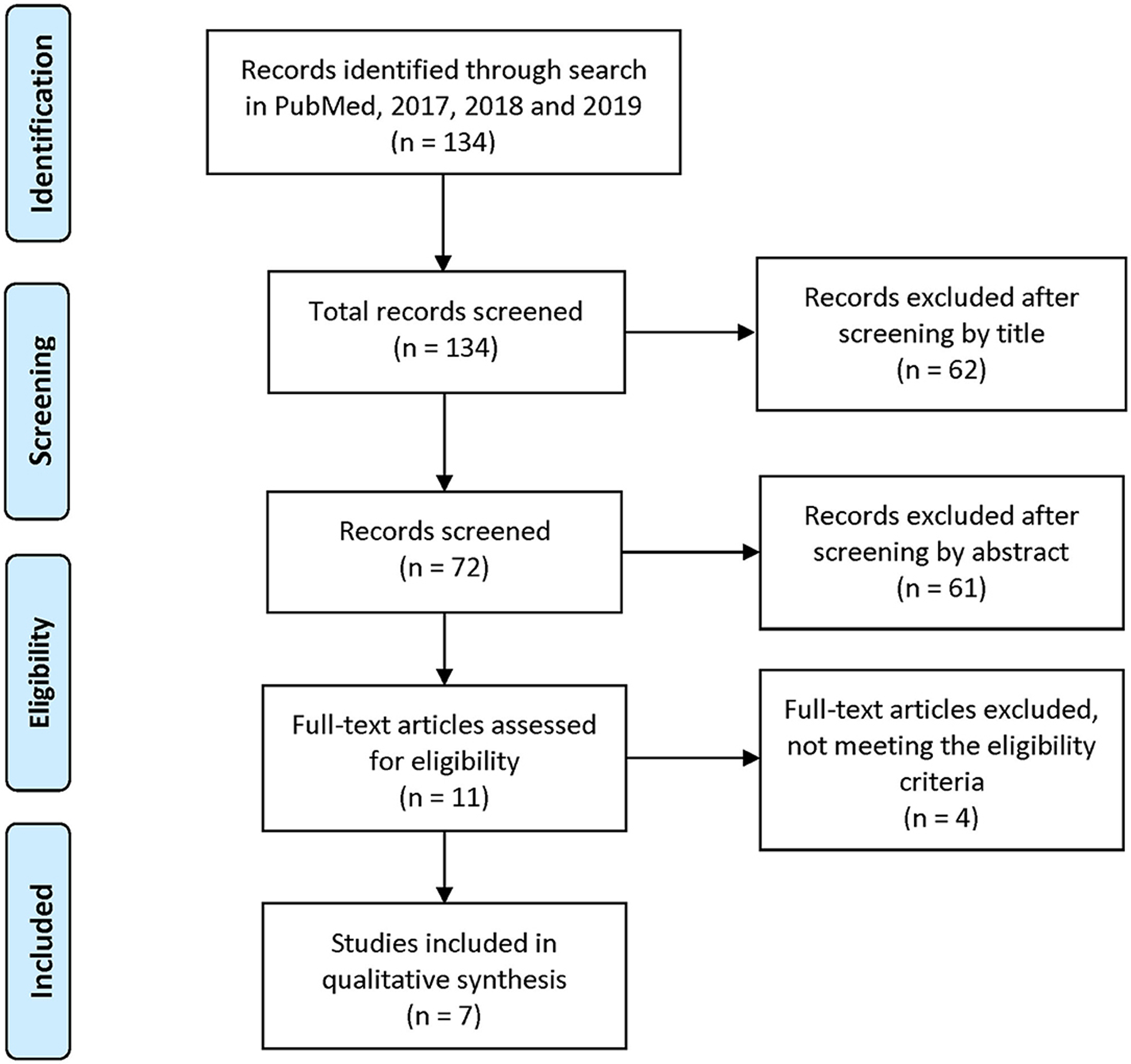
PRISMA 2009 Flow Diagram. Adapted from Moher et al. (2009).

Reviewers 1 and 2 each coded for the full set of variables (see Data Items). Subsequently, articles were reviewed and filtered, based on the inclusion/exclusion criteria listed in Eligibility Criteria. Figure 1 details the PRISMA search summary. Articles meeting the full inclusion criteria were reported.

### Data Items

Using an iterative process, Reviewers 1 and 2 identified the variables, and came to an agreement on final variable set. Articles were categorized by the following variables: keywords, article type (review, evaluation, research, protocol, other); inclusion of HIV+ individuals; study population; geographical location; technology mode(s) (i.e., SMS/testing, web app, online portal, etc.); intervention type, instrumentation; purpose or primary focus; main study outcomes; behavioral model(s); consideration of equitable access; privacy, confidentiality, and security considerations; and strengths, weaknesses and future directions, as stated by the article authors.

### Synthesis of Results

Applying our inclusion and exclusion criteria to randomized controlled trials and protocol articles resulted in a total of seven (*n* = 7) articles to review. Results were synthesized by evaluating sociodemographic characteristics of the study population, characteristics of the intervention and intervention outcomes, using the number of articles as the unit of analysis. Outcomes were reported separately for quantitative (*n* = 5) and qualitative (*n* = 2) study findings. Tables 1–3 summarize these findings. Table 4 summarizes how equity/equitable access, privacy and confidentiality was addressed for each study we reviewed.

## RESULTS

Here we summarize study characteristics, study population characteristics and mHealth system characteristics. We describe both published interventions and published protocols. and synthesize intervention quantitative outcomes and qualitative evaluations of the interventions. We then summarize our results for the research questions, i.e., models/frameworks, equity/equitable access and privacy/confidentiality). We end with results reported by the studies we reviewed, and the findings, gaps and areas for improvement resulting from our scoping review. Analyses are based on the individual articles as the unit of analysis.

### Study Characteristics

A total of 134 articles were initially identified, based on the search of the Medline database. After screening for titles and abstracts, 72 articles remained. From the review of these 72 articles for inclusion and exclusion criteria and extraction of data items for each of them, seven (7) articles—five (5) quantitative and two (2) qualitative—remained that met full inclusion criteria.

#### Sociodemographic Characteristics of Study Population

Analyzed variables for the seven (7) articles we reviewed are listed in Data Items. Three studies were conducted in the U.S., two in Africa, one in Europe, and one in China. The studies’ populations were diverse, including women, children, youth and older adults. The intended (direct) population for the majority of the studies (*n* = 6) was PWLH. The direct (target) population for one (1) study (Yè et al., 2018) was community workers, who provided services to PLWH—the *indirect* population.

Marent et al. (2018a)’s *EmERGE* intervention, designed for a multilingual and diverse population across five different European countries (Belgium, Croatia, Portugal, Spain, United Kingdom), included a sample of 97 patients in HIV care. The majority (65%, n = 63) were men who have sex with men (MSM).

The *PositiveLinks* application, used in both (Dillingham et al., 2018; Flickinger et al., 2018) studies, was tested with a cohort of 77 PLWH attending a rural, U.S. Ryan White-funded clinic. In the U.S., Ryan White funding supports HIV primary care for uninsured and underinsured patients. Participant characteristics included: 64% men, 49% Black non-Hispanic, with more than half reporting incomes below 50% of the Federal poverty level.

The *China Adherence Through Technology Study* (CATS) intervention enrolled 120 participants see (Sabin et al., 2015). Sabin et al. (2018) reported qualitative results on 20 participants who completed the treatment arm of the intervention. The CATS trial, conducted in a region in China experiencing a heroin epidemic, reported that 70% were male, 35% were injection drug users and 18% were alcohol consumers.

The target (direct) population for the *MOS@N* application was providers of HIV-related care and treatment services to the *indirect* population of PLWH. Yè et al. (2018) equipped 62 community workers–10 HIV/AIDS facilitators and 52 healthcare “godmothers” (former birth attendants)—with free mobile phones to interact with the MOS@N mobile application in a rural region of Burkina Faso. The community workers, who received the messages, were tasked with delivering appointment reminders to pregnant women living with HIV (their children), and to other PLWH. Yè et al. (2018) reported that 40% of the region did not own a mobile phone and connectivity was weak. The indirect population of the study was *n* = 2,051 persons, with- or at-risk of- HIV. Authors reported that 90% of community workers were illiterate, and, when surveyed, the majority reported having used a mobile phone for the first time during the training.

*SmartLink* is an mHealth app that was tested with 345 PLWH across 5 sites in South Africa (Venter et al., 2019). The app was designed to improve linkage to HIV care for young newly diagnosed PLWH. Participants were receiving care in public health sites (clinic, community health center, hospital) in Johannesburg. Worldwide, South Africa has the highest number of persons living with HIV (Avert, 2018). Venter et al. (2019) reported the following: ~65% women, 66% over 30 years old, 96% completed secondary school and 47% were employed full time. Thirty six percentage were originally from neighboring Zimbabwe, tended to have achieved higher levels of education and qualified for employment in Johannesburg.

#### mHealth System Characteristics

The forms of technology varied and included websites or portals, online community message boards, phone apps and SMS/texting. The mHealth SMS/texting systems were designed primarily to deliver message reminders for HIV-related appointments and medication alerts. A few addressed barriers to care, such as stigma and alcohol use. Table 1 summarizes each study’s intervention(s), study population, geographical region, and technology delivery mode by author and year published.

### Intervention Description

#### Published Interventions

The interventions reported in these articles were diverse in scope and in mode of delivery. The summary is provided in Table 1. The mHealth components included bi-directional SMS/text messages to individuals, messages triggered from pill counters, participation (postings) in an online message board, and access to online resources. Generally, the mobile technology was used for appointment and medication reminders, access to- or delivery of- healthcare information and education, and communication for social support. In all cases, texting was the delivery mode for appointment and medication reminders and alerts.

Marent et al.’s (2018a) *EmERGE* is a platform that supports multiple online sites. These sites bring together a patient information portal and enable social interaction between patients and providers. The platform was evaluated to support self-management to HIV care in Europe. The system will include SMS/texting reminder messages and will support bi-directional messages between patient and providers.

Dillingham et al. (2018) reported on *PositiveLinks*, an app designed to improve retention in care and clinical outcomes for PLWH. The app included the following features: tailored educational resources; daily queries of stress, mood and medication adherence; quizzes; appointment reminders; and a community message board (CMB). SMS/texting was the modality for reminder messages, bi-directional communication between patient and providers, and bi-directional messages that elicited information to tailor the system.

Flickinger et al. (2018) was the second article to report on PositiveLinks, with a focus on the influence of the CMB on perceived HIV stigma. For the CMB, individuals participated anonymously—initiating new conversations or responding to older conversations. The PositiveLinks team, eventually, initiated conversations on HIV topics and general well-being. Stigma was targeted as a known modifiable mediator of retention in care. PositiveLinks is also a resource for social support and community acceptance, with the potential to influence participant’s perception of stigma.

Sabin et al.’s (2018) *CATS* intervention tested personalized SMS/texting message reminders for medication adherence. Reminders were triggered when a Wireless Pill Container (WPC) was not opened 30 min beyond the prescribed dose time. During monthly clinic visits, participants received detailed reports, which documented their previous month’s adherence.

Yè et al.’s (2018) *MOS@N* intervention consisted of five training modules delivered to community workers on how to engage patients in care. MOS@N featured interactive voice response used to develop modules for patients’ management. As previously described, MOS@N community workers received automated SMS/text messages reminders to call patients and remind them of upcoming appointments.

Venter et al.’s (2019) *SmartLink* smartphone app sought to engage younger, newly diagnosed PLWH into care by providing laboratory results (CD4 and VL) securely and rapidly. The results were visually color-coded and scaled to distinguish normal values from abnormal values. SmartLink also provided a short explanation of the results and guidance for next steps. Laboratory values were linked from centralized site to the app.

#### Published Protocols

One published protocol, of the 134 articles we reviewed, met the inclusion criteria. Hightow-Weidman et al.’s (2018) protocol described the efficacy trials of several interventions developed by Emory University’s Center for Innovative Technology (iTech), including *YouThrive*, a web-based app to promote engagement in care, antiretroviral therapy adherence and viral load suppression for young men having sex with men (YMSM) living with HIV. The *YouThrive* app consists of: (a) peer-to-peer communication; (b) HIV care engagement SMS/text messages; (c) mood and cART adherence self-monitoring; (d) goal setting; and, (e) tailored cART and HIV informational content. Gamification techniques were used to promote sustained engagement. The detailed protocol was outlined in a separate manuscript (Horvath et al., 2019), published subsequent to our PubMed database search.

#### Intervention Outcomes

Interventions reported their outcomes as an improvement to adherence in HIV-related outcomes (*n* = 2) and as a reduction of personal-level barriers (*n* = 3) to HIV care adherence—barriers such as HIV stigma and alcohol use. Both approaches were taken as well (*n* = 2). To assess improvement in HIV care, adherence to appointment and medication uptake was measured (see Table 1).

Dillingham et al. (2018) (*PositiveLinks*) summarized their results as retention in care and visit constancy (minimum number of required visits over a period of time), with two secondary outcomes: improved HIV viral load and CD4 count. Retention in care, using the HRSA-1 definition, improved from 51 to 81% at 12 months (*p* < 0.0005). Dillingham et al. demonstrated significant differences. From baseline (22% with high visit constancy) to 12 months (51% visit constancy; *p* < 0.001). The mean CD4 count increased significantly (522 cells/mm^3^ vs. 614, *p* < 0.001), and the mean VL decreased significantly (23,682 copies/mL vs. 13,890, *p* < 0.002).

Flickinger et al. (2018) (*PositiveLinks*) compared HIV stigma scores from baseline to 12-month follow-up, 102.94 ± 18.26 to 98.73 ± 15.08, as a result of stigma-related postings (discussions) to the CMB. There was a trend toward reduced stigma, with a mean change of −3.9 (CI: 8.1, 0.2), which was not statistically significant (*p* = 0.060). However, when stratified, men experienced a significantly greater drop in stigma compared to women (7.1 ± 14.9 vs. 1.3 ± 13.8; *p* < 0.05).

Yè et al. (2018) (*MOS@N*) assessed intervention effectiveness among multiple clinical sites randomized to treatment or control, comparing pre- and post-intervention for key indicators that served as a proxy for HIV adherence—adherence to medical appointments and prevention of mother-child transmission of HIV. Other indicators such as adherence to health maintenance and follow-through on health-related referrals (e.g., high risk pregnancy and contraception care referrals) were also reported. Improvement in HIV care was demonstrated for the intervention regions, compared to the control regions. A significant decrease over a three-year period in loss to follow-up was reported as well—from 10% (2013) to under 1.6% (2016, *p* < 0.0001).

Venter et al. (2019) assessed linkage to care by capturing HIV-related laboratory monitoring, as surrogates to determine linkage to care. Laboratory values were recorded in the system, and values recorded between 2 weeks and 8 months from enrollment were counted as linkage to care. Statistically significant differences were found among youth/young adults (18 to 30 years old), comparing the control to the intervention group (31.9% vs. 53.0%, *p* < 0.01). Improvement was sustained for up to 16 months post-enrollment (50.7% vs. 69.9%, *p* < 0.02). Other variables analyzed like gender and viral load suppression were not statistically significant.

### Outcomes for Qualitative Evaluations

Here we summarize the data source and salient themes reported for the two studies reporting qualitative findings, as they relate to known barriers and facilitators to HIV care adherence and the barriers / facilitators of technology to facilitate HIV care adherence from the literature we reviewed. We synthesized Marent et al. (2018a) (EmERGE) and Sabin et al.’s (2018) (CATS) findings and categorized personal-level barriers and facilitators to HIV care adherence, and barriers and facilitators of technology as a vehicle for the intervention. Marent et al. (2018a) conducted workshops and semi-structured interviews with PLWH, providers and technology developers. Sabin et al. conducted in-depth interviews with PLWH enrolled in the treatment arm of their intervention.

Reviewers 1 and 2 independently analyzed the content of the two articles for participant-level barriers and facilitators. Using a thematic analysis approach (Braun and Clarke, 2006), content was reviewed recursively, coded manually and instances and constructs were further reduced to major themes. Reviewers consulted with each other and came to agreement on the relevant themes.

We found the following salient themes across the two studies: (a) taking personal responsibility for one’s own health condition and maintaining routines supports adherence, and digital platforms (reminders, access to clinical data, access to providers) facilitate these processes; (b) social support, counseling and good patient-doctor relationship promote adherence, and digital platforms (community groups, additional contact with providers) can facilitate these interactions; (c) HIV-related stigma is a concern; and, (d) digital platforms ease the burden of HIV stigma (fewer visits, anonymity). Participants cited the following disadvantages of technology. Technology can: (a) interfere with a person’s autonomy (e.g., “controlled” by technology); (b) interfere with the patient-provider relationship (e.g., face-to-face interactions are needed); and (c) interfere with different aspects of life (e.g., not enough privacy to attend to the technology at work; alerts interfere with other tasks). Findings are summarized in Table 2.

Flickinger et al.’s (*PositiveLinks*) study was unique in that it focused on the personal-level barrier of HIV-related stigma only. The authors evaluated content (postings) on the CMB, where overall 21% of the content was stigma related. The content was categorized as intrapersonal—negative content that described the participant’s internalized experiences of stigma—or interpersonal—negative content that described stigma experienced within relationships with others. Overcoming stigma was categorized as positive. Acknowledging self-image was categorized as positive intrapersonal content, and other positive content that addressed relationships with others was categorized as positive interpersonal content. In terms of overcoming stigma, intrapersonal positive experiences were reported in 31% of instances, including positive reframing of HIV positivity status (18%) and affirming self-worth (12%). Interpersonal positive experiences to overcome stigma were reported in 22% of instances, including finding true friendship/love/family (10%); positive past experience with disclosure (9%); and positive anticipated experience with disclosure (3%).

### mHealth Behavioral Models, Equity, Equitable Access, Privacy and Confidentiality

#### Behavioral Models

We assessed whether the mHealth interventions were grounded in one or more behavioral models or frameworks. Behavioral models were stated explicitly in 29% (*n* = 2) of the seven (7) studies we evaluated. However, only 14% (*n* = 1) cited a behavioral model for change. Findings are described here and summarized in Table 3.

Yé et al. applied the Technology Assessment Model (TAM) to their MOS@N intervention to assess acceptance of mobile technologies by HIV community workers responsible for reminders to PLWH. TAM considers perceived usefulness and perceived ease, as defined by Davis (1989). Perceived usefulness is the degree to which a person believes that using a particular system would enhance his or her performance (e.g., job performance). Perceived ease is the degree to which a person believes that using a particular system would require little effort. These two constructs, together, are determinants of technology-related behavior (Davis, 1989). However, we found no evidence that a behavioral model for change informed the design of MOS@N.

According to Hightow-Weidman et al., gamification techniques were used to develop their YouTHRIVE system, designed to engage YMSM living with HIV. “Gamification,” however, was not well-defined in the protocol. Multiple definitions have been applied to describe “gamification.” We found that YouTHrive aligns most closely with Huotari and Hamari’s (2012) definition: “A process of enhancing a service with affordance for gameful experiences in order to support user’s overall value creation” (Huotari and Hamari, 2012). Further investigation into YouTHrive in Horvath et al. (2019) showed that YouTHrive was adapted from Thrive With Me (TWM)—a peer-support, tailored self-monitoring ART adherence intervention grounded in the previously described IMB model (Amico et al., 2005).

#### Equity and Equitable Access

Equity/equitable access prevent differential healthcare outcomes for population subgroups, when compared to the general population (Solar, 2007). To ensure equitable access, one or more factors such as socioeconomic status (i.e., gender, education, income), differential exposure (risk factors, barriers), differential vulnerability (an individual’s disadvantaged state), differential healthcare outcomes, and differential personal consequences should be considered. As studies for this review were selected based on the inclusion criteria of a treatment adherence component, we therefore did not evaluate whether healthcare outcomes related to the mHealth intervention.

There are specific considerations related to technology that ensure equitable access. Intervention should consider adequate cellular and Internet coverage, sophistication of the mobile device to meet minimum requirements of the mHealth system and accommodations for low levels of general, health or technology-related literacy (Jack and Mars, 2014). Equity and equitable access, addressed in five of the studies, and privacy and confidentiality, addressed in six of the studies, are summarized in Table 4.

Yè et al.’s (2018) MOS@N intervention was designed specifically for an underserved population—women and children (under 5 years old) in the country of Burkina Faso. To leverage unequal access to services, community workers, many of whom did not own a phone, were provided free mobile phones to remind clients of upcoming appointments. Community workers were tasked with informing participants of the various HIV-related service, as well. Furthermore, community workers’ low literacy levels were considered in the design of MOS@N.

Marent et al.’s (2018a) EmERGE intervention included patient-community partners in various European cities, trained to facilitate interviews in the local European language. This accommodated participants who were not fluent in English. In addition, purposive sampling techniques, like proactively reaching out to women, ensured that a diversity of patients were represented in the qualitative phase of their study.

PositiveLinks, reported by both Dillingham et al. (2018) and Flickinger et al. (2018), was designed for ease of accessibility to rural and vulnerable patients in the U.S. PositiveLinks accommodated low literacy levels and inconsistent or lack of mobile phone access by providing a free smartphone to all participants. A tutorial on app usage was provided as additional education, as well. Although the app accommodated low literacy individuals, a few subjects (*n* = 4) (Dillingham et al., 2018) with extremely low literacy levels (lower than 4th grade) were excluded. Participants’ literacy levels were assessed using the Wide Range Achievement Test (WRAT-4).

The SmartLinks intervention (Venter et al., 2019) was designed for young adults under the age of 30 living with HIV—a population of high HIV transmission risk in South Africa. The app accommodated for both the English or Zulu language and communicated to participants in “simple” language. Laboratory values were visually color-code for easy interpretation. As the app was designed for young adult PLWH, there were no accommodations for older persons or for women, in fact pregnant women were excluded from participation. Venter et al. did explain, however, that participants were provided technical assistance by study staff.

#### Personal Privacy

Privacy is the right of an individual to keep his or her information from unlawful and improper collection and disclosure (U.S. Department of Health and Human Services, 2009; Staunton et al., 2019). For the purposes of this review, personal privacy, the protection of personal information—e.g., HIV status, legal name, drivers’ license number, and other identifying information—is distinguished from data privacy. Data privacy is reviewed separately in section Data Privacy.

Personal privacy was addressed in four (4) of the 7 studies we reviewed—Marent et al.’s (2018a) EmERGE, Dillingham et al. (2018), Flickinger et al.’s (2018) PositiveLinks, Sabin et al.’s (2018) CATS and Venter et al.’s (2019) PositiveLinks. Commonalities across the studies included the fact that personal privacy was incorporated into the mHealth system design (i.e., Dillingham et al., 2018; Flickinger et al., 2018; Venter et al., 2019), or addressed qualitatively, when participants expressed their concerns about personal privacy guarantees of digital technologies (e.g., Dillingham et al., 2018; Marent et al., 2018a; Sabin et al., 2018). Table 4 details specific examples.

#### Data Privacy

Data privacy is the ability to guarantee that patients’ data will be protected against both intentional and unintentional exposure (World Health Organization, 2015). Specifically, we consider the protection of digital information such as usernames passwords, online postings, browsing history, text messages and other digital communications, as data privacy. We found that data privacy was addressed explicitly in the PositiveLinks’ intervention (Dillingham et al., 2018; Flickinger et al., 2018), which encrypted mobile phones. Furthermore, to access the application, participants were required to log into a password-word protected system, and postings to its CMB were anonymous. Authors reported (see Table 4) that the PositiveLinks application is compliant with the U.S. Health Insurance Portability and Accountability Act (HIPAA), which guarantees data privacy. Data privacy was implied in Yè et al. (2018), who reported that the MOS@N developers followed “guidelines” for health-related mobile interventions. Data privacy was addressed in Marent et al. (2018a), whose focus group participants expressed concern that cloud-based systems would not protect their data. SmartLinks’ password- and personal identification number (PIN) ensured security of the app data.

#### Confidentiality

Confidentiality, which is the protection of health information from illegal or inappropriate disclosure (U.S. Department of Health and Human Services, 2009; Staunton et al., 2019), was embedded in the design of the PositiveLinks application (Dillingham et al., 2018; Flickinger et al., 2018). The PositiveLinks application is U.S. HIPAA-compliant, which guarantees confidentiality. Additionally, access and participation in the PositiveLinks CMB was via anonymous usernames, devoid of identifying information. The CMB was monitored to assess whether a breach of confidentiality had occurred. Participants were educated on the importance of anonymity when posting. During Marent et al.’s (2018a) focus groups, which were conducted to inform the EmERGE design, participants expressed concern that cloud-based systems would not protect information. Table 4 summarizes how confidentiality was addressed in each of the articles. SmartLinks modeled their app after the type of security employed with banking apps. Username, password, personal PIN number ensured confidentiality of health data.

### Findings, Gaps, and Areas for Improvement

We conducted a mixed-methods review, where we analyzed both quantitative and qualitative study outcomes. To answer our research questions, we used individual articles as the unit of analysis. To report on the most complete data available, we sought out additional, digital sources to complement information not available in the source articles.

#### Findings: Research Question 1

Our findings indicate that the majority of articles (*n* = 6; 86%) of the 7 articles did not report whether the HIV adherence interventions were developed under an established framework, nor were we able to verify this through other digitally published sources. Hightow-Weidman et al. specified only that “gamification” was the foundation of their YouTHrive intervention. Upon further investigation, we found that the IMB model was the framework for developing the gaming techniques for YouTHRive (Whiteley et al., 2018). We summarized findings in Table 3. We did count the TAM model described in Ye et al., although this model was the basis for measuring mobile technology acceptance among the HIV community workers, and not to develop the intervention.

#### Findings: Research Question 2

Strategies to address privacy or confidentiality were documented in the majority (*n* = 6; 86%) of the 7 studies we reviewed. However, only slightly over half of the studies (*n* = 4; 57%), namely Marent et al. (2018a) (EmERGE), Dillingham et al. (2018), and Flickinger et al. (2018) (PositiveLinks) and Venter et al. (2019) (SmartLinks) addressed both privacy and confidentiality. Results were summarized in Table 4. Other reviews (Burrus et al., 2018; Purnomo et al., 2018) emphasized the need for these considerations at each stage of development and implementation, and also to involve PLWH when designing the technology security features (Purnomo et al., 2018).

#### Findings: Research Question 3

The third research question this study sought to answer is what measures were taken to address or ensure equity and equitable access across different population subgroups. We found the majority (5 of the 7 studies, 71%) explicitly addressed equity and equitable access for PLWH. Equity was assessed according to the W.H.O.’s indicators of socioeconomic status, exposure or vulnerability (i.e., risks or barriers) and differential personal consequences (World Health Organization, 2010). Our evaluation also included technology-related factors that ensure equitable access, such as cellular and WiFi coverage, the ability of the mobile device (e.g., cellphone) to meet minimum requirements for use with the technology, and accommodations for low-literacy and low technology literacy individuals (Jack and Mars, 2014). Table 4 summarized the study findings that answered this question.

#### Findings Compared to Frameworks

Despite the lack of documentation regarding behavioral frameworks, we did find that (100%) of the four studies with quantitative results demonstrated significant HIV-related outcomes, i.e., improved visit attendance (Dillingham et al., 2018; Yè et al., 2018; Venter et al., 2019), improved HIV biological markers (Dillingham et al., 2018) and lessening of personal level barriers (Flickinger et al., 2018). Many used other approaches to design their interventions, namely Iterative processes that built on prior success, or contracting a commercial app development company. Other authors have proposed that traditional approaches of intervention testing and evaluation, like RCTs and implementation studies, can take years until completion and are not compatible with the rapidly evolving field of digitizing interventions (Mohr et al., 2013).

## DISCUSSION

The search and screening process identified seven (7) qualifying articles, which included four (4) interventions, one (1) protocol article and two (2) qualitative evaluations. The seven articles collectively reported on six different interventions designed to improve HIV care adherence, where one or more components were delivered via SMS/texting. SMS/texting reminders and alerts were stated features of four (4) published interventions—MOS@N, an intervention designed for the community workers in Burkina Faso who provided services to PLWH; CATS, an adherence app for PLWH in China; PositiveLinks, an adherence app for PLWH in the U.S., and SmartLInk, an app to link younger PLWH to care in South Africa. The PositiveLinks intervention app was described in two articles. We also reported on the protocol that described YouTHrive, a multi-feature (including SMS/texting reminders) web-based app designed for young MSMs in the U.S., and the formative, qualitative work completed to inform the design of EmERGE, a multi-service portal to support European PLWH.

### Primary Objective

Our primary objective was to evaluate certain ethical dimensions of digitized interventions designed to improve outcomes for PLWH. We mapped these onto Research Questions 1, 2, and 3. We reported on intervention theoretical frameworks/models, and whether privacy, confidentiality, equity and equitable access were addressed in these interventions.

#### Research Question 1

We found only one of seven (14%) of the articles we reviewed documented a theoretical framework or model. Previous reviews also noted incomplete or lack of documentation on models and processes for mHealth adherence Interventions (Catalani et al., 2013; Hightow-Weidman et al., 2015; Bauermeister et al., 2017). Models and frameworks are important considerations for technology-driven interventions in order to evaluate fidelity of the intervention execution (Bauermeister et al., 2017), gauge the success of the intervention overall and determine which specific components are successful (Hightow-Weidman et al., 2015; Bauermeister et al., 2017), consider combinations of multiple interventions (Muessig et al., 2017), facilitate tweaking of intervention to maximize use and effectiveness (Bauermeister et al., 2017), and finally create interventions that are quickly adaptable to change (Catalani et al., 2013). Models and frameworks facilitate intervention target, modification and evaluation.

Despite the limited information describing intervention models or theories (Hightow-Weidman et al., 2015), we found all (100%) of the six studies reporting quantitative results demonstrated one or more significant, HIV-related factors—improved visit attendance (Dillingham et al., 2018; Yè et al., 2018; Venter et al., 2019), improved HIV biological markers (Dillingham et al., 2018) and lessening of personal level barriers (Flickinger et al., 2018). We found authors of the studies we reviewed described other approaches to design their digital HIV interventions. Dillingham et al. and Flickinger et al.’s PositiveLInks was developed as a result of an iterative process. Venter et al. procured an app development company to build SmartLinks. It has also been proposed that the traditional models of intervention testing and evaluation, such as randomized control trials (RCT) and implementation studies are not the most efficient, cost-effective approaches to behavioral intervention technologies and are “fundamentally incompatible” with this rapidly evolving field (Mohr et al., 2013).

#### Research Question 2

Four of seven (57%) articles addressed privacy and confidentiality. For this review, we chose to distinguish personal privacy from data privacy, and reported on three concepts independently: personal privacy (e.g., HIV status, online postings), data privacy (digital information such as username, passwords, browsing history) and confidentiality (protection of health information). This approach emerged in the process of analyzing the qualitative studies, when it was revealed that the overarching concerns of the users enrolled in the studies we reviewed were related more to how the program, software or app protected their personal information like their HIV status. Users overwhelmingly expressed their concerns about security of their information and data (Marent et al., 2018a; Sabin et al., 2018), and text messages were sometimes intrusive (Marent et al., 2018a).

Other reviews reported similar concerns, that users of HIV digital interventions worried about the security and privacy of their information, and that alert messages were sometimes intrusive (Purnomo et al., 2018). Other authors have organized the concepts of privacy and confidentiality differently. In their commentary on digitized interventions (Burrus et al., 2018), for example, categorized security issues as the following: methodological, e.g., the minimal data required for the functionality of the application, technical, e.g., software application security, and procedural—who has access and how data breeches are handled. While there may be varying approaches to organizing privacy, confidentiality and security issues of digitized interventions, the basic premise holds, which is that healthcare providers and consumers of healthcare are equally concerned about the protection of information and data. Similar to what was reported in other reviews (Burrus et al., 2018; Purnomo et al., 2018), we did not find that privacy and confidentiality was addressed during the different stages of development and implementation, nor was it clear whether PLWH were involved in the security designs (Purnomo et al., 2018).

#### Research Question 3

Five of the seven (71%) articles we reviewed addressed equity and equitable access for the PLWH their interventions were designed for. For the studies that addressed equity and equitable access, the measures that were taken varied and were addressed at different points along the design-implementation-analysis continuum. During the design phase, input was elicited from a purposive and representative sample of participants, and apps accommodated bi-lingual and low-literacy individuals. At enrollment phase, the intervention targeted geographically hard to reach populations. For study implementation, free mobile devices were provided. Finally, at data analysis phase, outcomes were stratified to test intervention effectives by demographic characteristics.

Digitized HIV adherence interventions inherently seek to improve outcomes for PLWH; yet, the findings of other recent studies suggest that these interventions could further widen the gap of accessibility. Older adults and ethnic minorities in the U.S., for example, are less willing and less likely to use these technologies (Marhefka et al., 2020).

Another consideration is that effective interventions will not only result in improved outcomes, but the intended population will benefit equally (Amankwaa et al., 2018). Cell phone SMS/texting continues to be the most widely used and inexpensive form of mobile communication—available even to basic mobile phones (Chan and Kaufman, 2011; Miller and Himelhoch, 2013; Amankwaa et al., 2018). Yet, those with limited personal resources or those living in rural or remote regions that lack adequate infrastructure to support mobile devices, may not have access to even a basic mobile phone. The approach taken in one study we evaluated, the MOS@N project, was to implement a mobile intervention that supported community service workers, who received reminders to then remind PLWH. In very low resource environments, such as rural regions in the developing world, the approach taken by MOS@N is a cost-effective solution to equalize access for PLWH.

### Secondary Objective

Our secondary objective was to assess the studies’ findings. We examined quantitative, adherence-related outcomes and qualitative reports of perceived facilitators and barriers to adherence to HIV care and facilitators/barriers of technology. In summary, adherence to care, for studies with a quantitative design, was assessed by quantifying adherence to appointment visits, loss to follow-up, or indirectly by measuring laboratory values (CD4 or VL). Barriers to HIV care (HIV stigma), improved as a result of exposure to the mHealth interventions.

Studies with a qualitative design reported findings that were heterogeneous. However, we synthesized these findings and categorized them as facilitators and barriers to HIV care, and facilitators and barriers of technology. Mobile technology was perceived to be a facilitator more frequently than a barrier. Taken together, qualitative and quantitative findings in the studies we evaluated supported the use of mobile health technology as a strategy to improve HIV-related outcomes for persons living with HIV/AIDS.

### Strengths of This Review

To our knowledge, this is the only review, to date, assessing theoretical frameworks and models, privacy, confidentiality, equity and equitable access of mobile HIV adherence interventions. We sought to describe the most recently published findings, with a focus on SMS/texting. SMS/texting is inexpensive and is a feature available to the simplest phones currently in use, compared to native smartphone/mobile device apps, or accessing online systems via a mobile device.

In the case of incomplete or missing information, we reviewed other published articles and electronic documentation to fill the information gaps. We sought to include the most complete information available to answer our research questions. For our methodology, we ran a standardized query to retrieve the maximum number of articles possible, and we followed an organized protocol for data retrieval and processing.

### Limitations of This Review

We acknowledge that our review has several limitations. First, studies were limited to publications within a 2-year period (January 2017–June 2019). Second, our search was limited to the Medline database; our intent was to report on published, recent developments in mobile HIV adherence interventions. Third, our inclusion criteria yielded a heterogeneous pool of studies—both quantitative and qualitative results, and both research and protocol articles.

Fourth, the seven articles describing the six interventions we reviewed spanned different geographical areas (Asia, Africa, Europe, North America). Our definitions for privacy, confidentiality and equitable access were derived from South Africa, the United States, and the World Health Organization. These concepts may vary broadly across different countries. These limitations may be a potential reason why the designers of the interventions did not incorporate similar definitions and guidelines, when developing their mhealth interventions. Finally, it is plausible that the gaps we identified in the articles that we reviewed were due to a lack of documentation on the part the authors, and not that these important considerations were omitted in the intervention designs.

## IMPLICATIONS FOR RESEARCH, THEORY, AND PRACTICE

Mobile interventions hold promise as an emerging paradigm to enhance engagement into care and improve health outcomes for persons living with HIV. Particularly in large clinics, rural settings, or clinics in under-resourced settings, where the services that are available cannot meet the demand, mHealth interventions may serve as a vehicle to address health care disparities (Muessig et al., 2017) and improve adherence to HIV antiretroviral therapy (Purnomo et al., 2018). However, the body of knowledge on the ethical dimensions of these interventions has not kept up with the growth in mHealth applications, particularly as they apply to marginalized populations (Jack and Mars, 2014). We embarked on this review to address specific ethical concerns that emerged in the literature: whether digitized interventions were based on established models or theoretical frameworks; whether the technology platforms guaranteed privacy and confidentially; and, whether developers considered equity and equitable access for persons living with HIV when designing these interventions.

Our scoping review identified several gaps in the recently published mHealth literature. Only one of seven (14%) of the studies we reviewed documented a theoretical framework or model as the basis of their intervention. We found four of seven (57%) addressed privacy and confidentiality, and five of seven (71%) considered- or accounted for- equity and equitable access for the PLWH their interventions were designed for. We also identified the following areas of improvement for digitized HIV adherence interventions.

First, the literature recommends for mHealth interventions to be grounded within one or more models or theoretical frameworks for behavior change (Hightow-Weidman et al., 2015; Bauermeister et al., 2017); yet, we found insufficient documentation of behavioral models / frameworks. An overwhelming majority of the articles reviewed were *not* grounded within a model or framework. Frameworks enable designers to evaluate the fidelity of intervention execution (Bauermeister et al., 2017), determine the components that are successful (Hightow-Weidman et al., 2015; Bauermeister et al., 2017), facilitate intervention refinement (Bauermeister et al., 2017) and, where appropriate, combine multiple interventions (Muessig et al., 2017). Established frameworks are also important to determine the appropriateness of the intervention for the target audience. As an example, adolescents are more often non-adherent to HIV care, and are, therefore, a target population for digitized interventions; yet, certain frameworks that are applied to adults may be developmentally inappropriate for this age group (Hightow-Weidman et al., 2015). Similar to what was noted in earlier reviews (Bauermeister et al., 2017), we found a paucity of documented information describing process details of the interventions we reviewed.

Second, privacy and confidentiality are critical requirements for digitized HIV care solutions (Labrique et al., 2013; Jack and Mars, 2014), which stems from HIV being a highly stigmatized condition. These considerations are needed at each stage of development and implementation (Burrus et al., 2018). We determined that just over half of the articles we reviewed addressed personal privacy, data privacy and confidentiality; furthermore, these features were very important to participants in the studies we reviewed—individuals may not disclose their HIV status to family, friends or to their place of employment (Taylor et al., 2020). HIV stigma remains a concern worldwide for PLWH, and this was corroborated with the qualitative study findings we assessed. Other reviews (i.e., Purnomo et al., 2018), reported similar findings, as well. In Europe, for example, PLWH expressed that the even healthcare providers lacked knowledge about HIV (Marent et al., 2018b). Of note, providers in that same study also expressed their concerns regarding technology security features (Marent et al., 2018a).

Finally, equity, which considers socioeconomic factors, risk factors, personal consequences (World Health Organization, 2010), and equitable access, which includes technology-related issues (e.g., Wi-Fi access, smartphone ownership, sufficient data plans) and literacy are important ethical considerations for digitized solutions, particularly among marginalized groups (e.g., immigrants, elderly). Literacy encompasses both general literacy and technology-related literacy (Jack and Mars, 2014). We found that only one-third of the articles we reviewed addressed or incorporated equity/equitable access for their technology-based intervention.

There are both benefits and drawbacks of digital approaches to health for PLWH (Marent et al., 2018a), and we found technology was perceived as a barrier among the PLWH interviewed in the studies we evaluated. Participants expressed that technology could interfere with their ability to take control of the own healthcare, interfered with the relationship with their provider (Marent et al., 2018a), and interfered with certain personal aspects of their lives, as well (Sabin et al., 2018). Furthermore, PLWH expressed that technology could not be trusted to protect their information and data, and the increased use of technology put them at a higher risk of unintended disclosure of their HIV status (Marent et al., 2018a). When surveyed, older, minority, rural-dwelling Americans were not willing to engage with these technologies, even with the prospect a mobile phone at no cost (Marhefka et al., 2020).

Digitized interventions remain a viable option to support HIV care, however, despite the challenges posed by the technology, PLWH embraced these digital platforms as facilitators for adherence to their HIV care. Our synthesis of the qualitative findings we evaluated revealed that digitized interventions enable individuals to take responsibility for their health care, maintain a routine, provide support and access to both medical providers and medical information, and improve the patient-provider relationship (Marent et al., 2018a; Sabin et al., 2018).

Our mixed methods scoping review determined that privacy and confidentiality remain a concern for PLWH and that provisions to accommodate literacy (e.g., general and technology), infrastructure, technology and other challenges (e.g., access to smartphones and Wifi) are important ethical considerations to equalize outcomes and equitable access for PLWH. Future studies should report the behavior change models/frameworks or the formative work upon which their interventions are based and document their process details. Given the importance of privacy and confidentiality to users of mHealth systems, clearly defined privacy/confidentiality provisions will strengthen the intended users’ trust in these systems. And finally, although users recognize the benefits of technology, technology is also perceived a barrier for users, and these are important design considerations for mHealth developers.

What remains unclear is the role of theoretical frameworks and behavioral models. We found that despite the lack of documentation of frameworks/models, all quantitative studies we reviewed reported one or more significantly positive outcomes. Frameworks, models and benchmarks facilitate intervention evaluation, and, until recently, clinical trials and implementation projects were the traditional mechanisms by which intervention success was measure; yet, the rapid growth and evolution of digitized interventions brings into question the utility of these traditional approaches. Future studies, including an extended systematic review will not only shed light on these findings, but also determine whether the field of digitized interventions may be hampered by a paucity of documentation related to theoretical frameworks and models and a paucity of process documentation of these interventions.

As the global community works toward achieving the UN goal of ending the HIV/AIDS epidemic in the year 2030, interventions are needed that deliver successful outcomes, that are based on an ethical foundation and address the specific needs of the population. In the worldwide arena of digitized solutions, privacy, confidentiality and equitable access persist as ethical concerns—from the perspective of both providers and the persons for whom these technologies are designed. Users are not just concerned about their protection in the digital world, it affects their level of trust in these technologies, even when security is guaranteed by the stewards of the technology.

Digitized interventions bridge the gap between a scarcity of resources for those who are in the greatest need, yet, these interventions may further exacerbate inequities and unequal access. In high income regions like the U.S., the vast majority (e.g., 90%) of the population owns a mobile phone with at least the basic features of SMS/texting. However, more than a third (37%) of the world population does not own a mobile phone; and even for those who do, poor infrastructure may limit access to consistent service. Designers of digital interventions for persons living with HIV must consider if the most ethical approach to the design was taken, who has been excluded from access and is the intervention reaching the segments of the population most in need, if the global efforts will lead to the end of the HIV/AIDS epidemic in the coming decade.

## Supplementary Material

Supplementary File 1

## Figures and Tables

**TABLE 1 |:** Summary of article type, study population, geographical location, intervention, and technology mode for mHealth articles published January 2017–June 2019 that met inclusion criteria.

	References	Article type	Study population	Sample size	Geographical location	Intervention	Technology mode
1.	Marent et al. (2018a)	Research, qualitative	PLWH	*n* = 97	Europe: Cities in Belgium, Spain, UK, Portugal and Croatia	Support self-management and support for HIV care	EmERGE: portal to websites, smartphone apps; etc.
2.	Hightow-Weidman et al. (2018)	Protocol	YMSM	N/A	USA	Peer to peer interaction, engagement SMS, cART self-monitoring, goal setting, tailored ART adherence self-monitoring	YouTHrive: Web app
3.	Flickinger et al. (2018)	Research, quantitative and qualitative	PLWH	*n* = 77	USA: Virginia	Postings to an online community message board (CMB)—stigma-related messages analyzed	PositiveLinks: app; participation in online community message board (CMB)
4.	Dillingham et al. (2018)	Research	PLWH	*n* = 77	USA: Virginia	Phone app: Reminders, educational resources and CMB	PositiveLinks: educational resources, appointment reminders and CMB
5.	Sabin et al. (2018)	Research, qualitative	PLWH	*n* = 20*	Asia: China	Patient tracking and reminders	CATS: Real time Wireless Pill Container—SMS reminders
6.	Yè et al. (2018)	Research, Quasiexperimental design	Pregnant women, Children, PLWH	*n* = 62 (community workers)*	Africa: Burkina Faso	Reminders sent to HIV facilitators’ phone, who then contacted patients	MOS@N: SMS reminder once a day
7.	Venter et al. (2019)	Research	PLWH	*n* = 345	Africa: South Africa	Phone app: lab results, reminders, educational resources designed for younger PLWH	SmartLink: lab results (VL and CD4) and explanations, appointment reminders

* Sabin et al.’s primary study included n = 120 PLWH; Yé et al.’s primary study included n = 2051 PLWH.

**TABLE 2 |:** Summary of facilitators and barriers to HIV care, identified by the target population (PLWH): qualitative findings from the mHealth articles reviewed.

	Marent et al. (EmERGE)		Sabin et al. (CATS)	
	Patient-level	Technology-related	Patient-level	Technology-related
FACILITATORS	Taking/keeping control over his/her condition supports adherence Maintaining routine/ regularity supports adherence; in turn, reminders may not be needed Community groups would support adherence Expertise of providers and having access to providers for questions (outside of clinic visits) could support adherence	The digital platform can provide access to clinical data (VL, CD4) Regular medical visits are a tiring routine for PLWH who would benefit from receiving data through a digital device HIV is a stigmatized condition;technology can reduce the number of face-to-face visits to clinic, making the patient “invisible”—protecting their privacy and confidentiality Digital platforms can promote closer relationships with providers and to protect the privacy of HIV patients Secure digital networks can safeguard confidentiality	Technology facilitated taking personal responsible for one’s health Maintaining a daily routine promoted adherence Social support promoted adherence Counseling provided strategies to overcome barriers	SMS/text message reminders promoted routine/regularity The supervisory nature of the intervention promoted adherence Adherence reports were generated by the system, which promoted positive patient-doctor relationship
BARRIERS	Patients experienced stigma from providers and general public, which was a primary concern Concerns regarding stigma prevented patients from disclosing HIV status to others	Technology may result in a “passive” patient—one who is “controlled” by the technology device Technology takes away from face-to-face exchanges and experiences with providers Patients with unstable HIV may require face-to-face consultations to discuss treatment plan Increased use of technology could lead to unintentional disclosure of health data Patients do not trust security of cloud-based systems; systems unfortunately cannot repay a loss, like a bank can replace stolen funds Real-time alerts may be intrusive to everyday life	Barriers were primarily job related: forgetfulness, no breaks, concerns about inadvertent disclosure to coworkers, no private place to take medication Stigma surrounding HIV was a concern Alcohol and substance use interfered with adherence	Cellphone alarms and alerts may be “annoying” when performing other tasks and activities

**TABLE 3 |:** Summary of behavioral models, instrumentation, primary focus, and main findings for mHealth articles reviewed.

	References; intervention	Behavioral model(s)	Instruments/measurements	Primary focus	Outcomes/main findings
1.	Marent et al. (2018a); EmERGE	Not mentioned	Qualitative data analyzed from co-design workshops and interviews were used to develop the application	Understand “ambivalence” in the process of testing the mHealth platform for HIV care of PLWH	Multiple dimensions of ambivalence: quantification, connectivity, privacy and instantaneity
2.	Hightow-Weidman et al. (2018); YouTHrive	Gamification techniques. Based on the Information, Motivation, and Behavioral Skills (IMB) adherence model (Whiteley et al., 2018)	Not mentioned	Improve ART adherence among YMSM	N/A
3.	Flickinger et al. (2018); PositiveLinks	Not mentioned	Berger Stigma Scale	Decrease measured HIV stigma among participants	Reduced stigma score but not statistically significant
4.	Dillingham et al. (2018); PositiveLinks	Not mentioned	HRSA-1 Retention in care measures	Assess longitudinal impact on retention in care and viral suppression	Improved retention in care and visit constancy
5.	Sabin et al. (2018); CATS	Not mentioned	WPC: 95% threshold over 3 months—optimal vs. suboptimal	Assess participants’ (intervention arm) insights into the intervention	Improved mean on-time ART adherence and greater likelihood of achieving “optimal” adherence
6.	Yè et al. (2018); MOS@N	Technology assessment model (TAM), to assess technology acceptance	Structured questionnaire to captured key health indicators; Standard technology acceptance questionnaire	Increased access to health care for PLWH; mHealth intervention was applied to community facilitators	mHealth intervention contributed to more equitable access to health care for PLWH
7.	Venter et al. (2019); Smartlinks	Not mentioned	Centralized universal study dataset to capture VL, CD4 and creatinine clearance	Improve linkage to care for newly diagnosed PLWH using an smartphone app	Youth aged between 18 and 30 years old showed higher linkage to care

**TABLE 4 |:** Summary of equitable access, privacy, confidentiality, and security aspects for mHealth articles reviewed.

	References	Equity/equitable access addressed?	Personal privacy, data privacy and confidentiality addressed?
1.	Yè et al. (2018)	**Yes:** Population focus was pregnant women and children. Free mobile phones provided. Also, community workers’ literacy levels considered	**Data Privacy:** Guidelines for health-related interventions using mobile phones were followed
2.	Marent et al. (2018a)	**Yes:** Interviews were conducted in English and local languages, sampling to recruit a diversity of patients	**Personal Privacy, Data Privacy, Confidentiality:** Some participants expressed they did not trust the security of cloud-based systems and fear these are a threat to the security of their information and data (Marent et al., 2018b)
3.	Hightow-Weidman et al. (2018)	**No:** Not addressed for the study to optimize engagement in care	Not addressed
4.	Flickinger et al. (2018)	**Yes:** Low literacy accommodations; cell phone not required; smartphone was provided	**Personal Privacy:** Participants were advised to not reveal personally identifying information on the Community Message Board (CMB) **Personal Privacy, Data Privacy, Confidentiality:** The app was password-protected. The CMB was secure; postings were anonymous
5.	Dillingham et al. (2018)	**Yes:** app featured educational resources; patients given Samsung, incorporation of modification for low literacy population. The app reduced disparate healthcare outcomes, as retention in care for African Americans did not differ significantly from white, non-Hispanic population	**Personal Privacy, Data Privacy, Confidentiality:** The app included remote ‘locate and wipe” functionality; app was password secured and HIPAA compliant; postings to CMB were anonymous (as described in Flickinger et al., 2018) **Personal Privacy, Data Privacy, Confidentiality:** Application featured improved security, given phones were encrypted and password protected
6.	Sabin et al. (2018)	**No:** Not addressed	**Personal Privacy:** Participants were concerned their HIV status could be revealed
7.	Venter et al. (2019)	**Yes:** app delivery language was English and Zulu, lab values were color-coded to easy distinguish abnormal values, and the app language was “simple”	**Personal Privacy:** “HIV”, “AIDS” or “healthcare” not referenced on landing page or app icons **Personal Privacy, Data Privacy, Confidentiality:** Application modeled after banking apps. Username, password and personal pin was assigned to protect health data
