# Supplementary material for: Digitized HIV/AIDS Treatment Adherence Interventions: A Review of Recent SMS/Texting Mobile Health Applications and Implications for Theory and Practice
Source: Front Commun (Lausanne). Author manuscript; Available in PMC 2021 Feb 26. (PMC7909469; doi:10.3389/fcomm.2020.530164)
Supplement: Supplementary File 1 [file NIHMS1671354-supplement-Supplementary_File_1.docx]

Medline Search Strategy

((("Text Messaging"[Mesh]

OR Text*[Text Word]

OR Short Message Service[Text Word]

OR Text Messages[Text Word]

OR SMS messag*

OR “Methods”[Mesh]

OR Interventi*[Text Words])

AND ("Telemedicine"[Mesh]

OR mhealth[Text Word]

OR Mobile health[Text Word]

OR Telehealth[Text Word]

OR eHealth[Text Word])

AND ("HIV"[Mesh]

OR HIV[Text Word]

OR AIDS Virus[Text Word]

OR “HIV Seropositivity”[Mesh]

OR HIV seropositivit*[Text Word]

OR AIDS seropositivity[Text Word]

OR HIV Antibody Positivity[Text Word])))

NOT(("Text Messaging"[Mesh]

OR Text*[Text Word]

OR Short Message Service[Text Word]

OR Text Messages[Text Word]

OR SMS messag*)

AND ("Telemedicine"[Mesh]

OR mhealth[Text Word]

OR Mobile health[Text Word]

OR Telehealth[Text Word]

OR eHealth[Text Word])

AND ("HIV"[Mesh]

OR HIV[Text Word]

OR AIDS Virus[Text Word]

OR “HIV Seropositivity”[Mesh]

OR HIV seropositivit*[Text Word]

OR AIDS seropositivity[Text Word]

OR HIV Antibody Positivity[Text Word]))
